# Methylenetetrahydrofolate reductase genetic polymorphisms and esophageal squamous cell carcinoma susceptibility: A meta-analysis of case-control studies

**Published:** 2013-04

**Authors:** Yang Shujuan, Zhang Jianxing, Chen Xin-yue

**Affiliations:** 1 Yang Shujuan, West China School of Public Heath, Sichuan University, Chengdu, China.; 2 Zhang Jianxing, West China School of Public Heath, Sichuan University, Chengdu, China.; 3 Chen Xin-yue, West China School of Public Heath, Sichuan University, Chengdu, China.

**Keywords:** Methylenetetrahydrofolate reductase C677T, Polymorphism, Esophageal cancer, Folate intake, Meta-analysis

## Abstract

***Objectives: ***Genetic factors and environmental factors play a role in pathogenesis of esophageal squamous cell carcinoma (ESCC). Previous studies regarding the association of folate intake and Methylenetetrahydrofolate reductase C677T polymorphism with ESCC was conflicting. We conducted a meta-analysis to investigate the association of MTHFR C677T and folate intake with esophageal cancer risk.

***Methodology: ***MEDLINE, EMBASE and the Chinese Biomedical Database were searched in our study. The quality of studies were evaluated by predefined scale, and The association of polymorphisms of MTHFR C677T and folate intake and ESCC risk was estimated by Odds ratio (ORs) with 95% confidence intervals (CIs).

***Results: ***Nineteen studies (4239 cases and 5575 controls) were included for meta-analysis. A significant association was seen between individuals with *MTHFR 677 CT *[OR(95%)=1.47(1.32-1.63)] and *TT* [OR(95%)=1.69(1.49-1.91)] genotypes and ESCC risk (p<0.05). Low intake of folate had significantly higher risk of esophageal cancer among individuals with CT/TT genotype [OR(95%)=1.65(1.1-2.49)], while high intake of folate did not find significant high risk of esophageal cancer among individuals with CT/TT genotype [OR(95%)=1.64 (0.82-3.26)].

***Conclusions:*** Our meta-analysis indicated the folate intake and *MTHFR 677CT/TT *are associated with the risk of ESCC, and folate showed a significant interaction with polymorphism of MTHFR C677T.

## INTRODUCTION

Esophageal squamous cell and adenocarcinoma are common malignancies worldwide^1^, which is the sixth most commonly occurring cancer and sixth most common cause of cancer-related death in the world.^1^ The five-year survival rate for all stages combined was 15.6% from 1996 to 2003, which was much lower than most of other cancer types (ACS, 2008). Esophageal squamous cell carcinoma (ESCC) is one of the most prevalent cancer in China, and it is estimated 250,000 cases are diagnosed annually. Possible risk factors for ESCC include cigarette smoking, alcohol drinking, hot-temperature food, low intake of vegetable, salted food, pickled vegetables, chronic mucosal irritation and a family history of cancer.^1^^-^^6^ Deficiency of nutrients, such as vitamins and microelements, was suggested to be associated with an increased risk for ESCC.^6^

Folate is a water-soluble vitamin and naturally found in green leafy vegetables, cereals, legumes and fruits.^7^ Deficiency of folate could induce defective DNA repair and chromosomal fragile site expression, leading to chromosomal breaks and micronucleus formation.^8^ Methylenetetrahydrofolate reductase (MTHFR) C677T in the gene encoding the MTHFR enzyme, which converts dietary folate to its active cofactor in Hcy catabolism, has been studies as candidate genetic risk factor for esophageal cancer.^9^ As T allele dose increases, this functional polymorphism causes a graded elevation in individuals with low dietary folate consumption.^10^

Therefore, several previous studies have investigated the association of MTHFR C677T and folate intake with esophageal cancer risk, but the results are conflicting.^9^^,^^11^^,^^12^ The variation of these results might be induced by difference in ethnicities, sample size, study design and background of patients as well as random error. Therefore, we conducted a systematic review to investigate the association of MTHFR C677T and folate intake with esophageal cancer risk by reducing random error and obtaining precise estimates for some potential genetic associations.^13^

## METHODOLOGY

We searched MEDLINE (from Jan. 1966 to Jan. 2011), EMBASE (from January 1988 to Jan. 2011), and the Chinese Biomedical Database (CBM; from January 1980 to Jan. 2011) by using the following search strategy for published papers: ‘esophageal squamous cell carcinoma’, ‘esophagus’, ‘oesophagus’, ‘carcinoma or cancer or neoplasm or tumour or tumor’, ‘Methylenetetrahydrofolate reductase’, or ‘MTHFR’. There was no restriction on the language of published paper. All references cited in studies and previously published review articles were retrieved for additional eligible studies. The eligible criteria for including studies were (1) a case-control study reporting an association between *MTHFR C677T* polymorphisms and ESCC; (2) original study and an available genotype or allele frequency of *MTHFR C677T* genotypes for estimating an odds ratio (OR) with a 95% confidence interval (CI). If the results of a study reported two or more times on the same patient populations, only the most recent and complete study was included in our study.

***Data extraction: ***Two reviewers independently evaluated the retrieved articles, and the disagreements were resolved by discussion. Data retrieved from selected articles was included. In case the data were insufficient or missing, we attempted to contact the authors of the articles in order to request the relevant data. From those studies which werefinally selected, we extracted the following data: first author’s name, year of publication, country of origin, numbers of cases and controls, genotype frequencies of MTHFR C677T.

***Quality score assessment: ***The quality of studies was evaluated by predefined scale in previous studies^14^ (Table-I). The quality score assessment criteria were evaluated by traditional epidemiological considerations and cancer genetic issues. The quality scores ranged from 0 to 15. Score<10 was defined as low quality, and score≥10 was defined as high quality.

**Table-I:** Scale for Quality assessment

*Criterion Score*	*Score*
*Source of cases*	
Selected from population or cancer registry	3
Selected from hospital	2
Selected from pathology archives, but without description	1
Not described	0
*Source of control*	
Population-based	3
Blood donors or volunteers	2
Hospital-based (cancer-free patients)	1
Not described	0
*Specimens used for determining genotypes*	
White blood cells or normal tissues	3
Tumor tissues or exfoliated cells of tissue	0
*Hardy-Weinberg equilibrium in controls*	
Hardy–Weinberg equilibrium	3
Hardy–Weinberg disequilibrium	0
*Total sample size*	
>1,000	3
>500 and <1,000	2
>200 and <500	1
<200	0

***Statistical analysis: ***Statistical analysis was conducted by using STATA Statistical Package (version 9, STATA, College Station, TX). The distributions of genotypes in controls were tested by Hardy-Weinberg equilibrium (HWE) using the Chi-square test. The association of polymorphisms of MTHFR C677T and folate intake and ESCC risk was estimated by Odds ratio (ORs) with 95% confidence intervals (CIs). The heterogeneity was tested by the Q-statistics with p-values < 0.1, and its possible sources of heterogeneity were assessed by subgroup analysis. If there was heterogeneity, the random effect model was used. Otherwise, a fixed-effect model was applied to obtain the summary OR and their 95% CI. One-way sensitivity analysis was performed to explore robustness of the results. All P values were two-sided and a P value of less than 0.05 was deemed statistically significant.

## RESULTS

***Characteristics of studies:*** Forty seven studies were initially identified after search, and 28 studies were excluded due to overlapping data and being without meeting the criteria. Finally, 19 studies (4239 cases and 5575 controls) were included for meta-analysis. The detailed characteristics of these studies are summarized in Table-II. Only two studies had high quality score, and the scores of other studies ranged from 7 to 10. Of the 19 case-control studies, 14 studies were conducted in China.

A significant association was seen between individuals with *MTHFR 677 CT *[OR(95%)=1.47(1.32-1.63)] and *TT* [OR(95%)=1.69(1.49-1.91)] genotypes and ESCC risk (p<0.05)*.* There was significant heterogeneity between studies regarding *MTHFR 677 CT *and *TT* (P<0.05).

Subgroup analysis was taken according to folate intake, which indicated low intake of folate had significantly higher risk of esophageal cancer among individuals with CT/TT genotype [OR(95%)=1.65(1.1-2.49)] (Table-III). However, high intake of folate did not find significant high risk of esophageal cancer among individuals with CT/TT genotype [OR(95%)=1.64 (0.82-3.26)]. No significant heterogeneity was found between studies (P>0.05). These results indicated folate had a significant interaction with MTHFR C677T.

**Fig.1:**
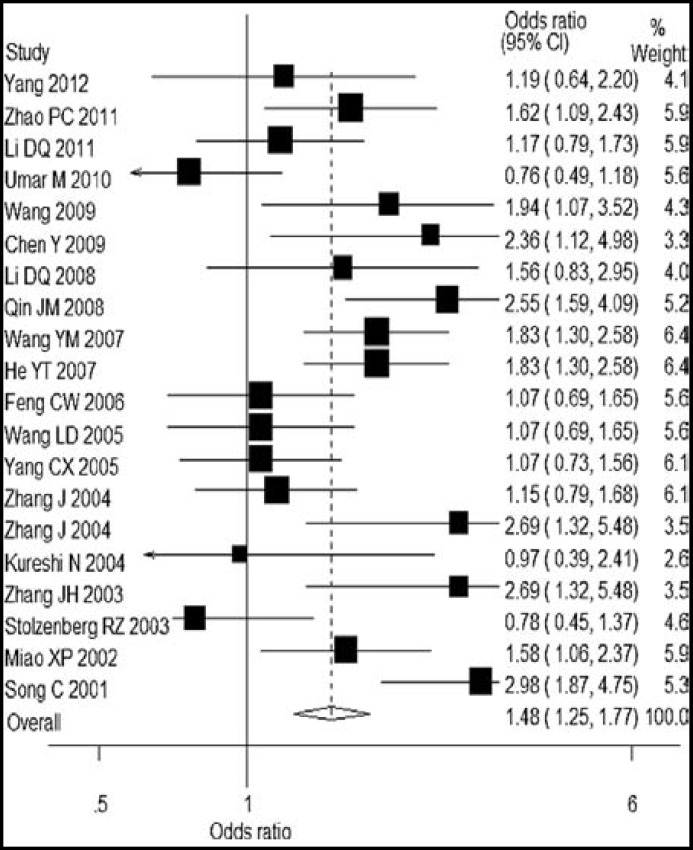
Publication bias on studies of* MTHFR 677CT* vs *CC*

**Fig.2:**
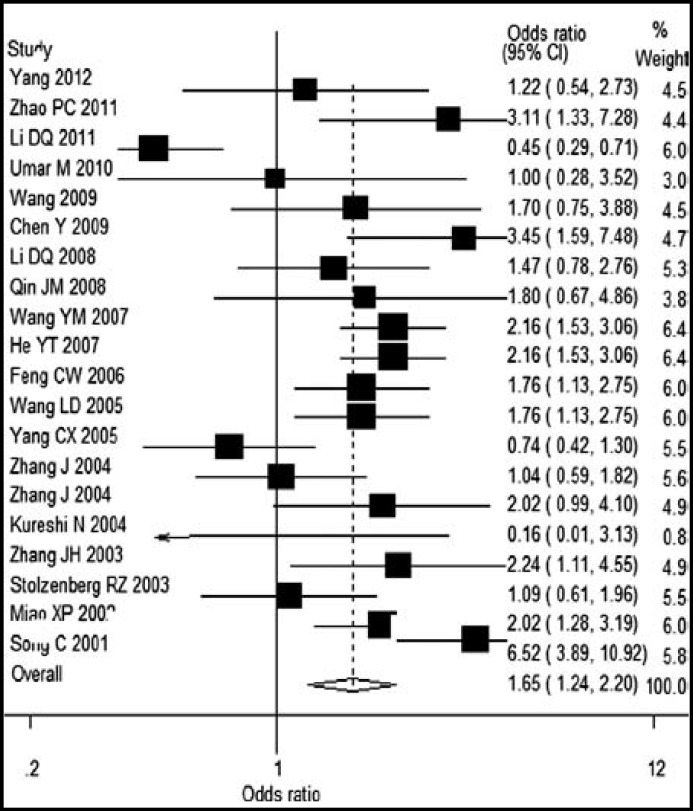
Publication bias on studies of *MTHFR 677TT* vs *CC*

A single study in this meta-analysis was deleted each time to reflect the impact of the individual data on the pooled ORs, and most of the results did not alter (Data not shown). Funnel plot an Egger’s test were used to assess the publication bias, and it provided evidence that there was no publication bias among studies regarding *MTHFR 677 CT, *but a significant publication bias was found in studies regarding *MTHFR 677* *TT *genotype(*P*<0.05). The shape A of funnel plots was asymmetrical (Fig. 1 and 2).

**Table-II:** Characteristics of studies of MTHFR C677T polymorphism and ESCC

*Study ID*	*County*	*Control source*	*Case*	*Control*	*Cases*			*Controls*			*P*_HWE_	*Quality score*
					*CC*	*CT*	*TT*	*CC*	*CT*	*TT*		
Chen Y 2009 (16)	China	Hospital	103	181	11	49	43	45	85	51	0.42	10
Feng CW 2006 (17)	China	Population	275	315	51	105	119	74	143	98	0.12	8
Zhao PC 2011 (18)	China	Hospital	155	310	68	74	13	179	120	11	0.09	9
Li DQ 2011 (19)	China	Hospital	226	246	112	113	45	95	82	85	<0.1	9
Li DQ 2008 (20)	China	Population	126	169	22	52	52	41	62	66	<0.1	10
Wang YM 2007(21)	China	Population	584	540	73	263	248	119	234	187	<0.1	11
Qin JM 2008 (22)	China	Population	120	204	60	53	7	170	59	11	0.06	11
He YT 2007 (23)	China	Population	584	540	73	263	248	119	234	187	<0.1	10
Song C 2001 (9)	China	Population	240	360	29	118	93	126	172	62	0.8	11
Wang LD 2005 (24)	China	Population	275	315	51	105	119	74	143	98	0.12	10
Yang CX 2005 (12)	Japan	Hospital	165	493	63	82	20	186	227	80	0.45	9
Zhang J 2004 (25)	German	Population	241	256	94	116	31	107	115	34	0.72	10
Zhang J 2004 (25)	China	Population	189	141	16	93	80	25	54	62	<0.1	10
Kureshi N 2004 (26)	Pakistan	Population	34	54	22	12	0	32	18	4	0.52	8
Zhang JH 2003(27)	China	Population	198	141	16	93	89	25	54	62	<0.1	7
Stolzenberg RZ 2003 (11)	China	Population	129	398	23	58	48	65	209	124	0.14	8
Miao XP 2002 (28)	China	Population	217	468	47	107	63	151	217	100	0.18	12
Umar M 2010 (29)	India	Hospital	208	223	155	48	5	155	63	5	0.63	13
Total			4239	5576	1008	1856	1375	1829	2353	1393		
Results of meta-analysis (Random effect model), OR(95% CI)			CT vs CC		1.47(1.32-1.63), P for heterogeneity: <0.05							
			TT vs CC		1.69(1.49-1.91), P for heterogeneity: <0.05							

**Table-III:** Subgroup analysis of *MTHFR C677T *polymorphism and ESCC

	*Cases*		*Control*	
*Folate intake *	*CC*	*CT/TT*	*CC*	*CT/TT*
Low folate intake				
Zhao 2011	21	21	37	26
Yang 2005	12	28	35	70
Qin 2008	41	81	37	33
Results of meta-analysis (Random effect model), OR(95% CI): CT/TT vs CC	1.65(1.1-2.49), P for heterogeneity: 0.41			
Moderate folate intake				
Zhao 2011	28	33	63	64
Results of meta-analysis (Random effect model), OR(95% CI)	-			
High folate intake	2.98(1.76-7.73)	0.25	3.35(1.84-6.12)	0.20
Zhao 2011	19	33	63	59
Yang 2005	50	151	74	237
Qin 2008	19	89	23	37
Results of meta-analysis (Random effect model), OR(95% CI)	1.64 (0.82-3.26), P for heterogeneity:0.13			

## DISCUSSION

Many epidemiologic studies which investigated the role of folate intake and *MTHFR C677T* for EC risk provided inconsistent results. Most of those studies involved few cases, and these few sample size limited the genetic effect reliabilty. Our meta-analysis recognized as an important tool to more precisely define the effect of selected genetic polymorphisms on risk of disease and to identify the potentially important sources of between-study heterogeneity. A previous meta-analysis in Asian population included 13 case-control studies which indicated *MTHFR 677 CT *and *TT* genotypes were significantly association with increased risk of esophageal cancer, especially in drinkers and smokers.^30^ However, this study did not explore the interaction between folate intake and MTHFR genotype. Therefore, we conducted an updated meta-analysis by critically reviewing 19 individual case-control studies on MTHFR C677T and folate intake with esophageal cancer risk. Compared with the last meta-analysis conducted in China by Fang et al, this updated meta-analysis included another 6 new case-control studies, and we explored the interaction between folate intake and MTHFR C677T. Our study showed that high intake of folate had a protective factor for esophageal cancer, and folate showed a significant interaction with polymorphism of MTHFR C677T.

Heterogeneity is a potential problems in the meta-analysis, and eliminating heterogeneity is an important factor during meta-analysis.^31^ In our study, we found there was significant heterogeneity between studies by using Q-statistics. However, after stratifying by the quantity of folate intake suggested folate was an important source of heterogeneity.

Previous studies have indicated folate mediates the transfer of one-carbon moieties both in the synthesis of nucleotides necessary for DNA synthesis, replication, repair and in DNA methylation reactions.^32^ These functions may play a critical role in carcinogenesis. Previous epidemiological studies have indicated an abundant intake of food stuffs full of folate could protect the development of various cancers.^33^ Ours study indicated that the folate intake was associated with a decreased risk of esophageal cancer, which proved previous hypothesis. Moreover, the activity of folate metabolic enzyme, such as MTHFR, are involved in the folate metabolic and DNA methylation process. As a key enzyme in folate metabolism, the product of MTHFR serves as the carbon donor for the methylation of homocysteine tomethionine, which is catalyzed by the enzyme MTR.^34^ The MTHFR gene is high polymorphic in the general population, the mutation of most common functional variant of 677C to T. This polymorphism results in an alanine to valine substitution, leading to a reduction in enzyme activity.^35^ The role of MTHFR in the folate metabolism decides the interaction between folate and polymorphisms of MTHFR, which was proved by our meta-analysis. Our study showed the MTHFR had strong risk of esophageal cancer in individuals with low intake of folate intake.

Possible limitations of this meta-analysis have to be considered in explaining our results. Firstly, most of the studies are conducted in China, and this could limit the power to find the difference in genotypes by different ethnicities. Secondly, publication bias may have occurred due to only published papers which were included in the meta-analysis. Thirdly, there might be misclassification during our study. Some controls in our study were selected from non-cancer inpatients, and some were selected from residents. Finally, there might be gene-environment interaction for esophageal cancer. However, we did not perform subgroup analysis due to lack of data on environmental factors. Further studies are warranted to interpreted this interaction.

In conclusion, our meta-analysis has indicated the folate intake and *MTHFR 677CT/TT *are associated with the risk of ESCC, and folate showed a significant interaction with polymorphism of MTHFR C677T. 
